# Detection at high prevalence of newlavirus (protoparvovirus) in the carcasses of red foxes

**DOI:** 10.1016/j.virusres.2022.198971

**Published:** 2022-10-17

**Authors:** Gianvito Lanave, Linda A. Ndiana, Francesco Pellegrini, Georgia Diakoudi, Barbara Di Martino, Giovanni Sgroi, Nicola D'Alessio, Violetta Vasinioti, Michele Camero, Marta Canuti, Domenico Otranto, Nicola Decaro, Canio Buonavoglia, Vito Martella

**Affiliations:** a University of Bari, Department of Veterinary Medicine, Valenzano, Bari, Italy; b University of Teramo, Faculty of Veterinary Medicine, Teramo, Italy; c Istituto Zooprofilattico Sperimentale del Mezzogiorno, Portici, Napoli, Italy; d Memorial University of Newfoundland, Department of Biology, St. John's, Newfoundland and Labrador, Canada

**Keywords:** Fox, Newlavirus, Parvovirus, Recombination

## Abstract

Wildlife conservation also relies on the study of animal virome. We identified the DNA of a novel fox protoparvovirus, newlavirus, with high (71%) prevalence in the carcasses of red foxes. On genome sequencing, high genetic diversity and possible recombination was observed, suggesting complex evolutionary dynamics in wildlife.

## Highlights

• Several novel parvoviruses have been identified in carnivores by deep sequencing.
• Newlaviruses (NLV) are a candidate *protoparvovirus* species found in foxes in 2021.
• Carcasses of red foxes were screened for NLV revealing a high (71%) prevalence.
• Marked genetic diversity was found in the VP1/VP2 gene among NLV strains.
• The evolutionary dynamics of NLV in fox and in other carnivores should be assessed.

## 1 Text

Parvoviruses, family *Parvoviridae*, comprise small, non-enveloped icosahedral viruses, approximately 20–30 nm in diameter, with a single-stranded linear DNA genome, 4–6 kb in length. Parvovirus genome possesses at both ends palindromic sequences that form hairpin structures, used for genomic replication, and two adjacent open reading frames (ORFs) which encode non-structural (NS) and structural (VP) proteins, respectively (Cotmore et al., 2019).

Parvoviruses infecting carnivores belong to the genera *Protoparvovirus, Bocaparvovirus, Dependoparvovirus* and *Amdoparvovirus*, subfamily *Parvovirinae*, and to the genus *Chaphamaparvovirus,* subfamily *Hamaparvovirinae* (Pénzes et al., 2020). With the exception of *Chaphamaparvovirus*, all the aforementioned genera have been detected in foxes (Ndiana et al., 2021; Martella et al., 2018; Bodewes et al., 2013; Melegari et al., 2019; Canuti et al., 2022, 2020).

The most important parvovirus in domestic and wild carnivores is represented by Carnivore Protoparvovirus 1 (CPPV-1), which includes canine parvovirus and feline panleukopenia virus (Tuteja et al., 2022).

In 2021 a new candidate protoparvovirus species, provisionally designated as Newlavirus (NLV), was identified with a high prevalence (up to 44%) in foxes from Canada. The virus displayed elevated genetic diversity at the VP1 gene level (70.5–87.8%) and was mainly identified in the feces and spleen. NLV was not detected in other animal species, and it was assumed that foxes could be the natural reservoir of the virus (Canuti et al., 2021).

The interest in wildlife research has been increasing in recent years as the result of the awareness that the health of human, animals and the environment are tightly connected, as postulated in the One Health envision. The study of wildlife viral diseases mostly relies on opportunistic sampling of carcasses and this constitutes an important resource for passive disease surveillance although potentially introducing biases into epidemiological studies (Nusser et al., 2008).

In the present study we performed a molecular screening for NLV on tissues collected from necropsied fox. A total of 100 retropharyngeal lymph node samples were collected from foxes (*Vulpes vulpes*), found dead by hunters, to perform routine necropsy procedures for diagnostic purposes. Sampling was carried out in different regions of Southern Italy (Calabria and Campania) from 2013 to 2016 (Ndiana et al., 2021). Samples were homogenized by a Tissue Lyser (Qiagen GmbH, Hilden, Germany) in 2 mL Eppendorf safe-lock tubes containing 1 mL of Dulbecco Minimum Essential Medium and a 4.8 mm stainless-steel bead (30 Hz for 5 min). Homogenized samples were subsequently centrifuged at 13,000 rpm for 3 min. DNA extraction was performed using QIAampDNA Blood & Tissue kit (Qiagen, Hilden, Germania). DNA extracts were subjected to a real-time PCR assay (qPCR) for the detection and quantitation of NLV DNA (Table 1). To confirm the presence of NLV, samples tested positive by qPCR were re-tested using a nested-PCR protocol for the amplification of a partial portion (328 bp) of the VP1 gene (Canuti et al., 2021) (Table 1). NLV copy numbers were calculated on the basis of standard curves generated by 10-fold dilutions of two pEX-A128 standard plasmids containing 500 bp of ORF2 region of NLV strain FX74 (GenBank accession no. MZ813280). The inserted gene was synthetized and cloned by Eurofins Genomics (Ebersberg, Germany). Log10 dilutions of standard DNA were evaluated simultaneously in order to obtain a standard curve for absolute quantification. All standard dilutions and unknown samples were tested in triplicate.

**Table 1:** List of oligonucleotides used in this study.

**Target gene**	**Assay**	**Primer/Probes**	**Sequence 5′−3′**	**Amplicon size (bp)**	**Reference(s)**
VP1	First round PCR	SlPV_ScF	AGACACTGACCAAGCACCC	355	(Canuti et al., 2021)
		SlPV_R1	GTACGATTGTCAAAATTGCCAG		
	Second round PCR	SlPV_ScF	AGACACTGACCAAGCACCC	328	
		SlPV_ScR	CTCAGGTTCCATTGGCTCG		
VP1	RealTime-PCR	NewlaF	TCCAAAGGCCGYGGRGCTAARAGA	82	This study
		NewlaR	CTAGTTGGCGYGCGTGYTTGAC		
		Newla-Pb	FAM-ACGACCCRGRAGAAGGACCTAGCG-BHQ1		
Full genome	Multiplex PCR	NWL1_F	AGGCCTCACTCACTAACTAACCAT	406	This study
		NWL1_R	TCCCCACTCTACCTGAATAAACCAT		
		NWL2_F	RGAAAATGGAGAAGAAGTGGAYATACC	424	
		NWL2_R	YAGGAAGTAATTAGCCACAAGYTCTGT		
		NWL3_F	AGAGCCATTGAAAATAATGAGTGGGT	418	
		NWL3_R	AGTCAGGGTCTTCAAGCATCCAAT		
		NWL4_F	AGAGAAATTGATCAACCTAGAGACATTGA	405	
		NWL4_R	RCCACTTTCYTTGTTGAGACARCACATCA		
		NWL5_F	CAGACTCTCTAGRTTYATGACTGCTTTTGA	438	
		NWL5_R	ACAGGTGTAGGYTCAATACTYTGAGAGCC		
		NWL6_F	ACTTTCCATTTAATGACTGTACCAGCA	424	
		NWL6_R	ATAGACGGYTCAGCCCAGTTCTCC		
		NWL7_F	TCCTGGTGATTTTGGVCTCATTGACA	437	
		NWL7_R	GGTARAGGAGCTTCAGGCATTACA		
		NWL8_F	ACGAACAGGAAACTTCTGGTTAACC	435	
		NWL8_R	AGCCTGGATGCAGCTCAAAGAT		
		NWL9_F	GACTGGGAACATGGTTTGGTTGAA	433	
		NWL9_R	ATAGAARTAAGGATTGAAGCCTGCTGC		
		NWL10_F	AGGCTACAAATACTTAGGGCCTGG	431	
		NWL10_R	CGTGYTTGACGYCGCTAGGTCCTT		
		NWL11_F	RTCCAAAGGCCGYGGRGCTAARAG	469	
		NWL11_R	TTGRAAGTCTGCTGGTGTCATCCA		
		NWL12_F	TACGCTTTGGCATTTTGACGGG	463	
		NWL12_R	ACATGGTCTCCAAGGAACRAAACCA		
		NWL13_F	ACTGCACTYATGYTARTTGCTGAAGACCA	475	
		NWL13_R	TGTCATYTTRCTGTAGGCCCARCCTTGT		
		NWL14_F	GCTTGGTGCACCRCCAAAAACAAM	418	
		NWL14_R	KTCTCTCATTCTTAGTTGGTTKGCWAGTGA		
		NWL15_F	YAACATGCTCACCAAAACWCAATATGA	400	
		NWL15_R	YAGTTTAACAAATAGTTGRCCTGGTAC		
		NWL16_F	AAYACCTACGGACCATTTGCAGCW	445	
		NWL16_R	AGCATACAATAWACCAAGTAAGTTACAGAGT		

In order to acquire the complete viral genome sequences of NLV strains, two multiplex PCR protocols amplifying sixteen PCR-tiling amplicons with a 400–475 bp size were designed, following an ARTIC-like strategy (Quick et al., 2017). Primer pairs were designed on the consensus sequence of NLV genomes recovered from the NCBI database (Table 1). The PCR assays were performed with TaKaRa La *Taq* polymerase (Takara Bio Europe S.A.S. Saint-Germain-en-Laye, France). All PCR products were pooled in equimolar ratios, quantified by Qubit dsDNA HS assay (Thermo Fisher Scientific, Waltham, MA) and used for library preparation and adapter-ligation with Genomic DNA by Ligation kit SQK-LSK110 (Oxford Nanopore Technologies, ONT, Oxford UK) following manufacturer's guidelines. Libraries were purified using Agencourt AMPure XP magnetic beads (Beckman Coulter™) and sequenced using flowcell flongle FLO-FLG001, R9.4.1 version adapted in a MinION Mk1C (ONT, Oxford UK) platform for 24 h. FastQ MinION files were subjected to quality control, trimming and reference assembly by Minimap2 and open reading frame (ORF) predictions, and annotations were performed in Geneious Prime software v. 2021.2.2 (Biomatters Ltd., Auckland, New Zealand). Sequence alignment was performed by MAFFT (Katoh et al., 2002). The phylogenetic analyses were performed using the maximum likelihood method implemented in MEGAX version 10.0.5 software (Kumar et al., 2018). Similarity plots were obtained using Simplot 3.5 (Lole et al., 1999).

**Fig. 1:**
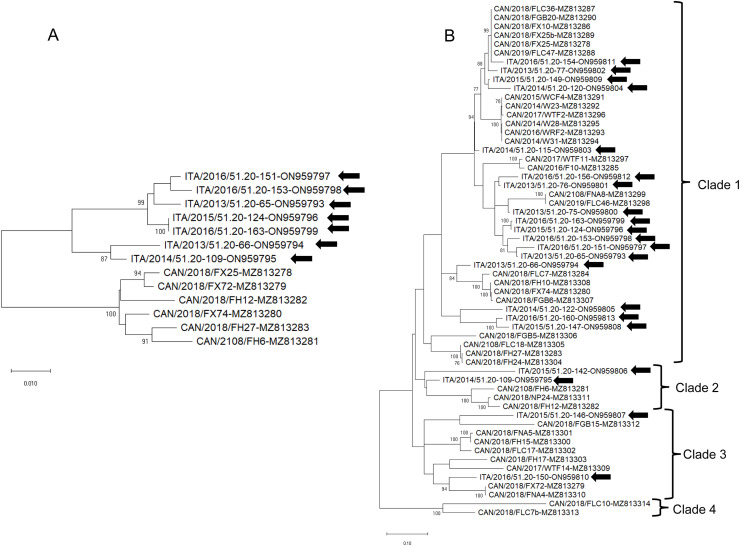
Full-genome-based unrooted phylogenetic tree of Newlavirus strains identified in this study and reference strains recovered in the GenBank database (panel A). VP1/VP2-based unrooted phylogenetic tree of Newlavirus strains identified in this study and reference strains recovered in the GenBank database (panel B). The Maximum Likelihood method and General Time Reversible model (six parameters) with a gamma distribution were used for the phylogeny. A total of 1000 bootstrap replicates were used to estimate the robustness of the individual nodes on the phylogenetic tree. Bootstrap values greater than 70% were indicated. Black arrows indicate strains detected in this study. Numbers of nucleotide substitutions are indicated by the scale bar.

**Fig. 2:**
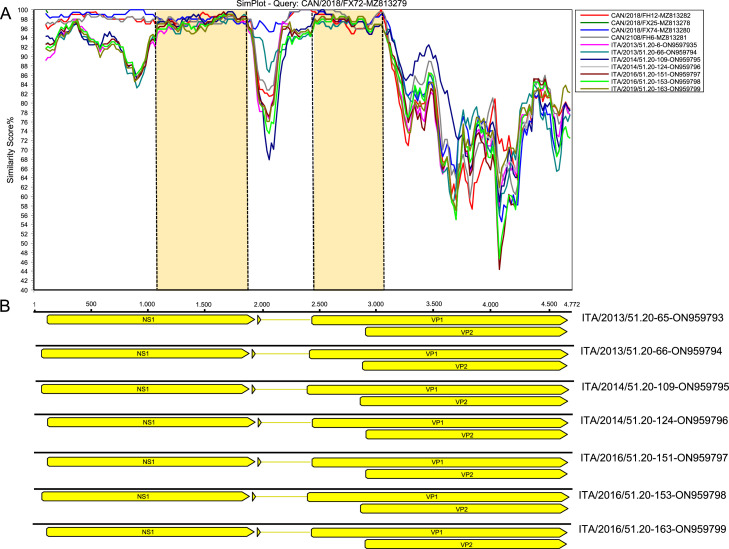
SimPlot analysis of full genome sequence of seven Newlavirus (NLV) strains identified in this study compared with the cognate NLV strain identified in Canada. Window size, 200 bp; step, 20 bp, Kimura (2 parameters) model. The vertical axis indicates the nucleotide identities between the query strain, CAN/2018/FX72 (MZ813279), and the other Italian and Canadian NLV strains, expressed as percentages. The horizontal axis indicates the nucleotide (nt) positions. Vertical dashed lines and light orange rectangles indicate the conserved regions (panel A). Schematic genome representations with *in silico* predicted ORFs of the seven Italian NLV strains (panel B).

NLV DNA was detected by qPCR with a 71% (71/100) prevalence. Viral load of NLV ranged from 2.3 × 10^1^ to 4.0 × 10^6^ DNA copies / ml (mean = 2.2 × 10^5^ DNA copies / ml, median = 4.9 × 10^3^ DNA copies / ml). A total of 21 positive samples, selected on the basis of viral DNA copies (≥ 10^3^ DNA copies/ml), were amplified by nested PCR and sequenced. Partial VP1 sequences (328 bp) obtained exhibited a nt identity ranging from 95.3 to 100.0 to cognate NLV strains previously identified (Canuti et al., 2021). The nearly complete genome sequences of 7 NLV strains (GenBank accession nos. ON959793—ON959799) and the partial VP1/VP2 gene sequences (918 bp) of other 14 NLV strains (ON959800-ON959813) were obtained. The genome size of the 7 Italian NLV strains identified in this study ranged between 4709 and 4772 nt and displayed an overall nucleotide (nt) identity ranging from 88.5% to 99.7% to other NLV strains retrieved from the GenBank database. The genome features of the identified NLVs comprised two major open reading frames (ORFs): ORF1 (1854 nt) encoding for NS1 protein (617 aa), and the ORF2 (2262–2268 nt), encoding for the VP1 (753–755 aa) and VP2 (588–590 aa) proteins, respectively (Table 2). The nucleotide alignment of the complete NS1 and the partial VP1/VP2 (663 bp) sequences of NLV strains identified in this report and cognate reference strains recovered in the GenBank database displayed an overall nucleotide (nt) identity ranging from 93.8% to 100.0% and 70.2% to 100.0%, respectively. Upon phylogenetic analysis, in the NS1 region the 7 Italian NLV strains clearly diverged from the Canadian viruses (Fig. 1A), whilst in the partial VP1/VP2 gene (663 bp) the 21 Italian NLV strains were intermingled with the Canadian NLVs in three distinct genetic clusters (Fig. 1B), a pattern which may imply recombination, a phenomenon not uncommon in parvoviruses (Shackelton et al., 2007). By SimPlot analysis, a highly conserved region was mapped in the middle portion of the NS1 throughout the alternative splicing site of the N terminal domain of the VP1 region (from nt 1100 to nt 1900), using as reference the NLV strain FX25 (MZ813278) (Fig. 2). Another highly conserved region spanned the VP1 unique region throughout the initial part of the VP2 (from nt 2500 to 3120), where the qPCR was targeted. Genome variation was higher in the spliced region and, in particular, in the VP2 coding region.

**Table 2:** Genomic features of complete and partial genomes of newlavirus (NLV) strains sequenced in this study.

**Sample ID**	**Accession**	**Size (nt)**	**NS1**		**VP1**		**VP2**			**Full-genome identity toreference sequences**		**NS1 gene identity toreference sequences**		**VP1 gene identity toreference sequences**		**VP2 gene identity toreference sequences**	
			**nt**	**aa**	**nt**	**aa**	**nt**		**aa**	**NLV strain #(accession nr.)**	**nt identity%**	**NLV strain**⁎⁎**(accession nr.)**	**nt identity%**	**NLV strain**⁎⁎**(accession nr.)**	**nt identity%**	**NLV strain**⁎⁎**(accession nr.)**	**nt identity%**
ITA/2013/51.20–65	ON959793	4772	1854	617	2268	755	1773		590	FX25 (MZ813278)	93.2	FX25 (MZ813278)	94.1	FX25 (MZ813278)	92.0	FX25 (MZ813278)	90.6
ITA/2013/51.20–66	ON959794	4758	1854	617	2265	754	1770		589	FX74 (MZ813280)	92.8	FX72 (MZ813279)	94.3	FX74 (MZ813280)	91.5	FX74 (MZ813280)	89.9
ITA/2014/51.20–109	ON959795	4744	1854	617	2262	753	1767		588	FH12 (MZ813282)	92.1	FX25 (MZ813278)	94.9	FH12 (MZ813282)	90.5	FH12 (MZ813282)	88.8
ITA/2014/51.20–124	ON959796	4769	1854	617	2268	755	1773		590	FX25 (MZ813278)	93.8	FH27 (MZ813283)	94.5	FX25 (MZ813278)	92.8	FX25 (MZ813278)	91.5
ITA/2016/51.20–151	ON959797	4768	1854	617	2265	754	1770		589	FX25 (MZ813278)	91.6	FX72 (MZ813279)	94.6	FX25 (MZ813278)	88.8	FX25 (MZ813278)	86.6
ITA/2016/51.20–153	ON959798	4709	1854	617	2265	754	1770		589	FX25 (MZ813278)	92.1	FX25 (MZ813278)	94.4	FX25 (MZ813278)	90.0	FX25 (MZ813278)	88.0
ITA/2016/51.20–163	ON959799	4771	1854	617	2268	755	1773	590		FX25 (MZ813278)	93.6	FH27 (MZ813283)	94.5	FX25 (MZ813278)	92.4	FX25 (MZ813278)	91.2
ITA/2013/51.20–75	ON959800	912*	–	–	912⁎⁎	304	879	293		–	–	–	–	FX25 (MZ813278)	91.1	FX25 (MZ813278)	90.7
ITA/2013/51.20–76	ON959801	912*	–	–	912⁎⁎	304	879	293		–	–	–	–	FX25 (MZ813278)	93.8	FX25 (MZ813278)	93.7
ITA/2013/51.20–77	ON959802	912*	–	–	912⁎⁎	304	879	293		–	–	–	–	FX25 (MZ813278)	97.3	FX25 (MZ813278)	97.3
ITA/2014/51.20–115	ON959803	912*	–	–	912⁎⁎	304	879	293		–	–	–	–	FX25 (MZ813278)	95.4	FX25 (MZ813278)	95.2
ITA/2014/51.20–120	ON959804	912*	–	–	912⁎⁎	304	879	293		–	–	–	–	FX25 (MZ813278)	95.5	FX25 (MZ813278)	95.4
ITA/2014/51.20–122	ON959805	912*	–	–	912⁎⁎	304	879	293		–	–	–	–	FX25 (MZ813278)	89.2	FX25 (MZ813278)	88.9
ITA/2015/51.20–142	ON959806	909*	–	–	909⁎⁎	303	876	292		–	–	–	–	FX72 (MZ813279)	84.7	FX72 (MZ813279)	84.2
ITA/2015/51.20–146	ON959807	906*	–	–	906⁎⁎	302	873	291		–	–	–	–	FX72 (MZ813279)	86.8	FX72 (MZ813279)	86.4
ITA/2015/51.20–147	ON959808	915*	–	–	915⁎⁎	305	882	294		–	–	–	–	FX25 (MZ813278)	88.5	FX25 (MZ813278)	88.0
ITA/2015/51.20–149	ON959809	912*	–	–	912⁎⁎	304	879	293		–	–	–	–	FX25 (MZ813278)	97.6	FX25 (MZ813278)	97.5
ITA/2016/51.20–150	ON959810	906*	–	–	906⁎⁎	302	873	291		–	–	–	–	FX72 (MZ813279)	90.7	FX72 (MZ813279)	90.4
ITA/2016/51.20–154	ON959811	912*	–	–	912⁎⁎	304	879	293		–	–	–	–	FX25 (MZ813278)	97.7	FX25 (MZ813278)	97.6
ITA/2016/51.20–156	ON959812	912*	–	–	912⁎⁎	304	879	293		–	–	–	–	FX25 (MZ813278)	92.2	FX25 (MZ813278)	92.1
ITA/2016/51.20–160	ON959813	915*	–	–	915⁎⁎	305	882	294		–	–	–	–	FX25 (MZ813278)	88.5	FX25 (MZ813278)	88.1

nt, nucleotides; aa, amminoacids

⁎ partial genome sequence

⁎⁎ partial VP1 sequence; # sequence with the highest identity on interrogation of GenBank database with BLAST.

Overall, the prevalence of NLV in foxes in Italy was higher than previously observed in Canada. In particular, considering only the lymph nodes collected from the head, in our study we observed a nearly 15-fold difference (71.0% versus 5.4%) (Canuti et al., 2021). This high detection rate of NLV in foxes could be accounted for by a pattern of persistent infection or, eventually, by reactivation of the virus in stressed/weakened animals, rather than being directly related to the death of the animals.

Finally, the high genetic diversity observed in the VP2 gene of NLV contrasts with the high genetic conservation of other protoparvoviruses, such as CPPV-1, where a few punctate mutations result in major biological changes (Ndiana et al., 2021; Tuteja et al., 2022), suggesting a long-term adaptation of NLV in foxes or intricated dynamics of circulation of virus variants among multiple susceptible hosts. Epidemiological studies in wild animals might help understand more in depth the pathogenic role, if any, of NLVs, and the evolutionary dynamics of these viruses in foxes and, eventually, in other animal hosts.

## Ethical statement

The authors confirm that the ethical policies of the journal, as noted on the journal's author guidelines page, have been adhered to. No ethical approval was required as this study was conducted on carcasses of animals found dead and submitted to routine necropsy procedures for diagnostic purposes. All experiments were performed in accordance with relevant guidelines and regulations.

## CRediT authorship contribution statement

**Gianvito Lanave:** Writing – original draft, Data curation, Software, Formal analysis. **Linda A. Ndiana:** Investigation. **Francesco Pellegrini:** Investigation. **Georgia Diakoudi:** Investigation. **Barbara Di Martino:** Methodology, Validation. **Giovanni Sgroi:** Resources. **Nicola D'Alessio:** . **Violetta Vasinioti:** Investigation. **Michele Camero:** Visualization. **Marta Canuti:** Supervision. **Domenico Otranto:** Resources. **Nicola Decaro:** Project administration. **Canio Buonavoglia:** Supervision. **Vito Martella:** Writing – review & editing, Supervision.

## Declaration of Competing Interest

The authors declare that they have no known competing financial interests or personal relationships that could have appeared to influence the work reported in this paper.

## Data Availability

Data will be made available on request.
